# Intravenous Endotoxin Challenge in Healthy Humans: An Experimental Platform to Investigate and Modulate Systemic Inflammation

**DOI:** 10.3791/53913

**Published:** 2016-05-16

**Authors:** James N. Fullerton, Elisabetta Segre, Roel P.H. De Maeyer, Alexander A.N. Maini, Derek W. Gilroy

**Affiliations:** ^1^Centre for Clinical Pharmacology, Division of Medicine, University College London

**Keywords:** Medicine, Issue 111, Endotoxin, immunology, lipopolysaccharide (LPS), systemic inflammatory response syndrome (SIRS), cytokine, sepsis, infection, translation, human experimentation, cell kinetics, immune function, physiology, critical illness

## Abstract

Activation of inflammatory pathways represents a central mechanism in multiple disease states both acute and chronic. Triggered via either pathogen or tissue damage-associated molecular motifs, common biochemical pathways lead to conserved yet variable physiological and immunological alterations. Dissection and delineation of the determinants and mechanisms underlying phenotypic variance in response is expected to yield novel therapeutic advances.

Intravenous (IV) administration of endotoxin (gram-negative bacterial lipopolysaccharide), a specific Toll-like receptor 4 agonist, represents an *in vivo* model of systemic inflammation in man. National Institutes for Health Clinical Center Reference Endotoxin (CCRE, *Escherichia coli* O:113:H10:K negative) is employed to reliably and reproducibly generate vascular, hematological, endocrine, immunological and organ-specific functional effects that parallel, to varying degrees, those seen in the early stages of pathological states. Alteration of dose (0.06 - 4 ng/kg) and time-scale of exposure (bolus *vs.* infusion) allows replication of either acute or chronic inflammation and a range of severity to be elicited, with higher doses (2 - 4 ng/kg) frequently being used to create a 'sepsis-like' state. Established and novel medicinal compounds may additionally be administered prior to or post endotoxin exposure to appreciate their effect on the inflammatory cascade. Despite limitations in scope and generalizability, human IV endotoxin challenge offers a unique platform to gain mechanistic insights into inducible physiological responses and inflammatory pathways. Rationally employed it may aid translation of this knowledge into therapeutic innovations.

**Figure:**
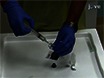


## Introduction

Inflammation of differing etiology, severity and duration forms a central component in the pathology of multiple diseases. Ranging from the critical illness states such as sepsis or trauma where severe, relatively short lived inflammation predominates, to chronic illnesses including type 2 diabetes mellitus, atherosclerosis and Alzheimer's disease where persistent low-level inflammatory tone is considered a pathologic factor, dysregulated inflammatory pathways are a key driver of global morbidity and mortality^1,2^.

Inflammation is initiated by the presence of pathogen-associated molecular patterns (PAMPs) or danger-associated molecular patterns (DAMPs) — commonly intracellular components released via tissue damage^3-5^. These conserved molecular motifs bind pattern-recognition receptors (PRRs) such as Toll-like receptors (TLRs) found on epithelial cells, endothelial cells and those of the innate immune system, to trigger common downstream intracellular signaling pathways^6^. Activation of the nuclear transcription factor nuclear factor κB, amongst others, leads to transcription of genes encoding pro-inflammatory cytokines that establish the inflammatory cascade^7^.

Inflammation induces both local and systemic effects designed to isolate and remove the primary insult. Local alterations include up-regulation of adhesion markers, chemokine release, vasodilation and increased vascular permeability to facilitate innate immune cell transmigration. The complement and coagulation systems are also activated. Systemically, multiple organ-specific physiological changes are observed, orchestrated via the neuroendocrine network. Significant increases in humoral mediators occur and the circulating cell profile radically alters. Failure of inflammation to cease and homeostasis to be reestablished may be due to persistence of the initiating stimuli or a failure of dedicated resolution pathways^8^. These potent mechanisms, often coordinated by lipid mediators may themselves be pathogenic^9,10^.

Investigation of the inflammatory response and these key regulatory steps in the clinical population is challenging. Demographic (age, sex, ethnicity), temporal (time of onset, duration of exposure) and clinical variance (type of initiating stimuli, severity of insult, co-morbidity burden, therapeutic intervention) impede access to key biological pathways. The traditional solution has been to use animal models. Despite affording multiple advantages, their fundamental similarity to human pathophysiology^11,12^ and the relevance of their output, has come under increasing scrutiny^13-15^. An alternative is to employ reductionist human models.

Developed in the late 1960s, intravenous (IV) endotoxin administration to man offers a vital platform through which to discover, delineate and potentially drug inflammatory pathways^16-19^. Endotoxin (used synonymously with lipopolysaccharide [LPS] in this article) is a potent TLR4 agonist that triggers the inflammatory cascade in a dose-dependent manner. Injected as either a bolus or infusion it may thus be used to model both low-level and moderate systemic inflammation of differing duration. The resultant short-lived inflammatory response permits assessment of a single component of the highly complex host-pathogen interaction that develops during bacterial infection. While not a model of shock, IV endotoxin challenges elicits responses that appear to replicate the early stages of infection. Eliminating inter-species translational barriers and accurately mimicking the clinical phenotype qualitatively if not quantitatively, it affords real-time interpretation of inflammatory influences, consequences and an opportunity to test interventions. Endotoxin may also be administered endobronchially^20^, intradermally^21^ or in combination with IV LPS^22^ to explore local inflammatory responses in different compartments. Less widely employed alternative methodologies include infusion of proximal pro-inflammatory cytokines such as tumor necrosis factor α (TNFα) and interleukin (IL) 6^19^. These will not be discussed further here.

## Protocol

University College London REC (UK) approved the study that generated the data presented in the representative results section (Reference 5060/001).

**ETHICAL APPROVAL:**

The majority of studies using IV endotoxin challenges have been conducted in healthy subjects. As this research has no health benefit, the importance of the study objectives should clearly outweigh any inherent risks. This is relevant when considering the interactions with novel agents used to modify inflammation and that have the potential to exaggerate host responses to endotoxin (*e.g., *fever, changes in blood pressures, symptoms).

Prior to the commencement of any study employing IV endotoxin, approval must be sought and obtained from an appropriate Research Ethics Committee (REC) / Institutional Review Board (IRB). IV endotoxin administration has been employed for over forty years to gain important mechanistic insights into human biology with no serious or lasting adverse effects to our, or other author's, knowledge^19^. Given the model's potential to yield vital information pertaining to multiple inflammatory conditions that are major causes of mortality and morbidity, we believe it is ethically acceptable to expose healthy volunteers and defined demographic or clinical groups to endotoxin, provided appropriate risk minimization strategies are in place. This may require independent outside review by qualified experts and or pilot studies to ensure safe conduct of the investigations, particularly when employing new agents and their interaction with endotoxin- induced inflammation.

### Preparation 

### 1. Determine the Demographic and Clinical Characteristics of Participants

**NOTE:** The majority of studies employing IV endotoxin challenge recruit healthy young male volunteers < 30 year old. Studies have additionally been undertaken on healthy women^23^, older volunteers^24,25^ and patient subgroups^26^ depending on the experimental question. Elderly participants (*i.e.*, greater than 60 years of age) may have a greater and more sustained response (higher fever, greater decrease in blood pressure), and interactions of endotoxin-induced inflammation with any co-morbid conditions or medications must be considered in the design of these studies. Please see discussion for further considerations in participant selection.

### 2. Recruit Participants 

1. Arrange suitable advertisement as required. **NOTE:** Financial compensation for participant’s time and inconvenience is commonly offered. This must be established prior to recruitment and approved by a REC/IRB.
2. Issue a REC/IRB approved Participant Information Leaflet (PIL) explaining the nature of the study, an overview of the protocol, expected side effects, and risks associated with IV endotoxin to respondents meeting inclusion criteria. **NOTE:** This should be issued in advance of consenting to allow an informed decision to be made to participate and for any questions or concerns to be addressed by the researcher.

### 3. Obtain Formal Informed Consent

1. Obtain written consent from all participants prior to undertaking any study-related procedure, including health screening.
2. Seek verbal confirmation of participant retention and understanding of the information in the PIL.
3. Discuss side effects of IV endotoxin administration. **NOTE:** At higher doses (2 - 4 ng/kg) these include rigors (chills), headache, photophobia, myalgia, arthralgia, nausea, and rarely, vomiting. Peak symptom intensity occurs around 1 - 2 hr post-injection, abating afterwards to baseline by 6 - 8 hr. No severe or sustained adverse effects secondary to endotoxin at these doses have been reported. Rarely, a volunteer may find the degree of symptoms unacceptable during the height of the response. The symptoms can be ameliorated with administration of paracetamol/acetaminophen or non-steroidal agents by mouth or IV (*e.g.*, aspirin, ibuprofen). Such agents may alter the inflammatory response and their use should be recorded.
4. Re-affirm the ability of participants to withdraw from the study at any point without giving an explanation.

### 4. Undertake a 'Health Screen' on Potential Participants

**NOTE:** This is to ensure the absence of undisclosed medical conditions that place them at greater risk of harm from IV endotoxin. As a secondary aim information relevant to the experimental question may be identified. 

1. Identify a suitable clinical environment and an appropriately trained medical professional to conduct the health screen.
2. Take a full clinical history including past medical and social histories, past and on-going medication/treatments, novel health foods or over the counter pharmaceuticals, allergy status and an enquiry into the presence of current symptoms that may suggest new or recent illness.
3. Perform a formal clinical examination of, as a minimum, the cardiovascular and respiratory system.
4. Arrange and review core investigations including routine observations (weight, heart rate, blood pressure, respiratory rate, oxygen saturations, temperature), blood work (full blood count, renal, hepatic and coagulation functional tests), and 12-lead electrocardiogram. **NOTE:** Additional tests may be required according to the experimental protocol.
5. Have the participants with unremarkable history, examination and investigations proceed to IV endotoxin challenge, dependent on REC/IRB-approved inclusion/exclusion criteria. **NOTE:** It is best practice for newly detected symptoms, signs or test abnormalities to be reported to and investigated by the participant’s routine physician. These individuals should be excluded until any concern has been appropriately addressed.

### 5. Inform Participants Proceeding to IV Endotoxin Challenge to; 

1. Notify the investigator of any new symptoms or illness. **NOTE:** This will normally preclude their entry into the study.
2. Ask the participants arrive at a pre-stated time and location. Advise the participants to wear comfortable clothes and bring entertainment.
3. Ask the participants to fast from midnight (clear fluids permitted).
4. Ask the participants to refrain from alcohol and caffeine for 24 hr prior to injection and/or a pre-defined period afterwards (optional and protocol dependent)。

### Procedure

### 6. Endotoxin Challenge (Prior to Endotoxin Administration)

1. Prepare a bed with the head at 45°. **NOTE:** Ensure this is located in an appropriate location to gain clinical assistance and resuscitation equipment. For instance, a Clinical Research Facility, anesthetic room or intensive care unit.
2. Ensure the required clinical and experimental equipment is accessible and functioning. **NOTE:** This includes a sphygmomanometer, thermometer, pulse oximeter and, if being employed, 3-lead cardiac monitoring. Verify completeness of local resuscitation equipment and accessibility of oxygen.
3. Reconstitute Clinical Center Reference Endotoxin
  1. Add 5 ml of Sterile Water for Injection, USP (SWI) to a previously un-reconstituted vial of CCRE using full aseptic non-touch technique (ANTT) throughout. Vials contain 10,000 endotoxin units (EU) (approximately 1 µg) in the form of a white, lyophilized powder.
  2. Place the vial of CCRE with 5 ml SWI added on a vortex shaker for 1 hr to ensure endotoxin adherent to the glass surface of the vial is fully dissolved. **NOTE: **CCRE does not go into solution readily, despite appearing to have fully-dissolved.
  3. Draw the correct weight-adjusted volume of endotoxin (now 2,000 EU/ml or roughly 200 ng/ml) into a 1 ml syringe. **NOTE: **Use glass or polypropylene syringes to minimize adherence (and loss) of endotoxin to equipment.
4. Participant
  1. Confirm consent to proceed with IV endotoxin administration. Enquire about new illness, symptoms and compliance with protocol specific instructions. **NOTE: **If there is medical concern or non-compliance endotoxin, delay the administration.
  2. Ask the participant to lie in the prepared bed, confirming they are comfortable. Ensure they have voided bladder and bowel.
5. Obtain vascular access.
  1. Insert an intravenous cannula under ANTT for the administration of endotoxin, and if included in the protocol, intravenous fluids.
  2. Place a second intravascular line for on-going blood draw to avoid repeated venous stab. Insert this intravenously and connect to a 3-way tap (with or without an extension) to facilitate blood draw and flushing. **NOTE:** Alternatively, for continuous blood pressure monitoring and sample acquisition, site an intra-arterial line. This requires greater expertise for insertion and there is an increased risk of local complications.
    1. For on-going blood draw using intravenous cannula, use an 18 gauge or larger bore cannula along with a siting in the antecubital fossa to avoid hemolysis and clotting. IV fluid administration will help maintain patency. **NOTE: **Selection of intravascular cannula insertion type and site should take place according to individual practitioner and participant preference. Intra-arterial lines are traditionally placed in the radial or femoral artery with prior instillation of local anesthetic (*e.g., *1% Lidocaine).
  3. Draw blood for baseline tests upon insertion of either the first or second line. Discard the first 5 ml of blood (occupying dead-space within the cannula and connectors) prior to obtaining blood for sampling. Flush the line with 10 ml of 0.9% sodium chloride after blood draw. **NOTE:** The volume of blood drawn and handling of the samples will be determined by the experimental protocol and local laboratory procedures.
  4. Attach clinical monitoring as appropriate (*e.g.*, 3-lead cardiac monitoring is usually employed with higher dose challenges 2 - 4 ng/kg).
  5. Record baseline routine clinical observations as stated in 4.4 on an appropriate vital sign chart. Subjective symptom scoring (*e.g.*, using visual analogue scores for headache, myalgia, *etc*.) should additionally be performed to monitor the participant experience.
6. Endotoxin Administration
  1. Flush the intravenous cannula with 0.9% sodium chloride to ensure the cannula is correctly sited and patent.
  2. Administer the pre-prepared, weight-adjusted dose of reconstituted CCRE via the same intravenous cannula. Inject the bolus (< 2 min) dose into a 3-way tap at the hub of the IV line.
  3. Flush with 10 ml 0.9% sodium chloride to ensure all CCRE enters the circulation. **NOTE:** To administer a continuous infusions inject the endotoxin into a known volume of diluent, *e.g.*, 100 ml of 0.9% sodium chloride to achieve a pre-determined concentration, and infuse the resultant solution at a set rate (volume/time).

### 7. Monitoring, Observation and Sample Draw

1. Monitor participants undergoing IV endotoxin for a minimum of 6 hr after bolus dosing or for the duration of an infusion. Have a qualified, experienced clinician perform this step.
2. Record and review vital signs and any clinical observations at a minimum of once/hr. Continuous monitoring such as 3-lead cardiac monitoring may additionally be undertaken. **NOTE:** Investigators should note that at higher bolus doses (2 - 4 ng/kg) vasovagal reactions may provoke cardiac pauses. These are most frequent 30 min to 2 hr after injection and are not malignant (see discussion for further details).
3. Undertake subjective symptom scoring at the discretion of the investigator.
4. Administer 2 - 3 L of crystalloid (for instance 0.9% sodium chloride or Hartmann's solution) over 6 - 8 hr after bolus administration of 2 - 4 ng/kg. **NOTE:** No acceptable standard exists for administration of the above mentioned fluids.  Safety of participants is paramount. This provides routine maintenance fluid and volume replacement (for the period kept nil by mouth and increased insensible losses due to raised body temperature and respiratory rate). They may also counter the risk of cardiac arrhythmias (see discussion).
5. Obtain pre-determined samples required to answer the experimental question. **NOTE:** These traditionally include, but are not limited to, blood and urine. The timing, number and volume of samples should be the minimum required to obtain accurate data and have been approved by the REC/IRB. More frequent sampling may be required at certain time points (*e.g., *every 30 min) to ensure peak (or trough) values are not missed. Calculations of blood volume taken must account for blood discarded during withdrawal of dead space.
6. Process samples as required.

### 8. End of Procedure

1. Have the attending clinician ensure that the participant's symptoms have settled and that their observations are trending to normal (all altered parameters, *e.g.,* elevated heart rate and temperature, demonstrating consistent reduction toward baseline values) prior to sanctioning the end of observation and subsequent discharge. **NOTE:** After bolus injection of 2 - 4 ng/kg CCRE symptoms normally fully abate by 6 - 8 hr. Individual observations follow overlapping but discrete time-courses. These have normally returned to baseline by 10 - 12 hr. Continuous infusion requires a longer period of observation as symptoms and vital signs will not subside immediately post cessation of endotoxin administration.
2. Remove all monitoring equipment and intravascular lines ensuring hemostasis.
3. Confirm the participant is happy to return home and has the contact details of investigator in case of any concern. **NOTE:** It is considered best practice to contact the participant the following day to check their health status and monitor for any sequelae.

## Representative Results

The diverse, multi-system consequences of IV endotoxin administration have recently been comprehensively reviewed and will not be discussed in full here (for full details see^17-19,27^). Instead a brief overview of the range and applicability of the model will be provided alongside primary data pertaining to the classic bolus-dosing regimen (2 ng/kg). A schematic diagram illustrating the timeline of such an experiment is provided in **Figure 1**.


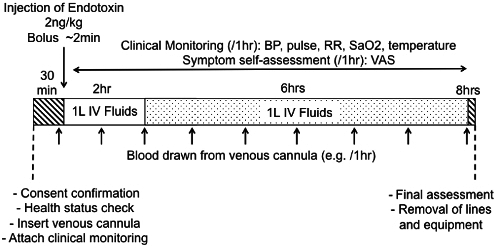
**Figure 1: Representative Schematic of Bolus IV Endotoxin Challenge Protocol. **After arrival, participant's consent to proceed should be confirmed along with their health status and protocol compliance (fasting, alcohol abstention,* etc.*). Following insertion of intravascular lines, attachment of monitoring and collection of baseline clinical observations and samples, a weight-adjusted dose of reconstituted CCRE is administered. Clinical monitoring for a minimum of 6 - 8 hr is mandatory. Samples, most commonly blood, may be taken at pre-selected time-points. Crystalloid intravenous fluids are commonly given, in this case 1 L over 2 hr followed by 1 L over 6 hr. Please click here to view a larger version of this figure.

The effect of IV endotoxin is dependent upon the dose selected and the method of administration (bolus *vs.* infusion). A bolus of 2 - 4 ng/kg reliably elicits the features of the systemic inflammatory response syndrome with raised core temperature, heart rate and white blood cell (WBC) count^16^. This is accompanied by the presence of humoral mediators of inflammation including pro and anti-inflammatory cytokines, acute phase proteins (such as C-reactive protein) (see **Figures 2** and **3**) and activation of the hypothalamo-pituitary axis^28^ and both the pro-coagulatory and fibrinolytic systems^29^. Metabolism in both central and peripheral tissues is altered and multiple organ-specific functional alterations elicited^19,30^. Participants experience a variety of 'flu-like' symptoms that peak in intensity around 1 - 2 hr and largely abate by 6 hr (see **Figure** **4**). The bolus 'high-dose' protocol induces a qualitatively consistent but quantitatively variable response in volunteers^31^.


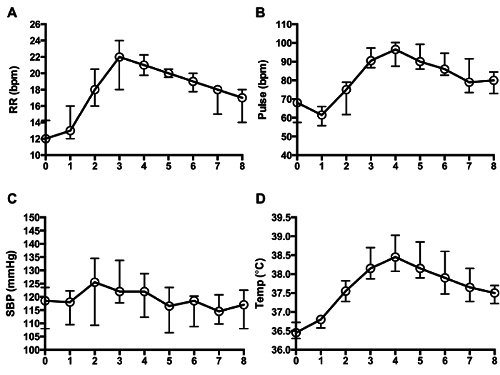
**Figure 2: Clinical Observation Profile. **Change in vital signs post-bolus administration of Clinical Center Reference Endotoxin 2 ng/kg (median with IQR, n = 10). Respiratory rate (RR, breaths per min, **A**), pulse (beats per min, **B**), systolic blood pressure (SBP, mmHg, **C**) and temperature (°C, via tympanic thermometer, **D**) are displayed. SBP did not fall due to administration of IV fluids, the small rise likely reflecting increased sympathetic outflow. Time is displayed on the x-axis (hr post-endotoxin administration). Please click here to view a larger version of this figure.


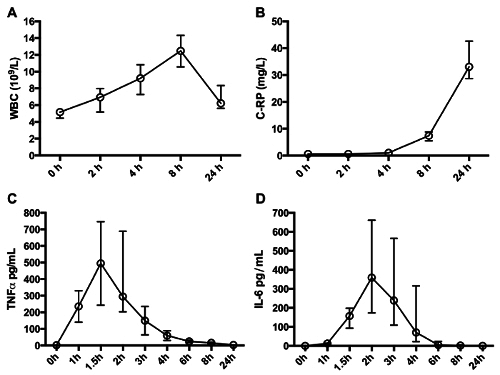
**Figure 3: Cellular and Humoral Response.** Consistent alterations in total leukocyte count, including transient neutrophilia, are observed (**A**) along with commonly used clinical indicators of inflammation such as C-reactive protein (**B**). Quantitatively varied, yet qualitatively conserved alterations in circulating plasma cytokine concentrations occur after IV endotoxin. TNFα (**C**) and IL-6 (**D**) are displayed as exemplars. All data post-bolus administration of Clinical Center Reference Endotoxin 2 ng/kg (median with IQR, n = 10. Cytokine concentration represents the mean of 2 technical repeats/individual at each time point). Please click here to view a larger version of this figure.


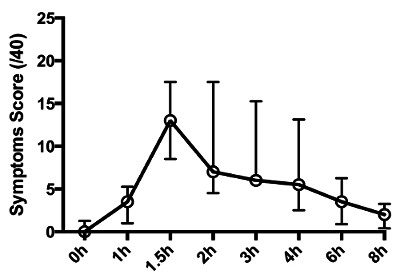
**Figure 4: Symptom Profile. **Cumulative symptom score recorded at pre-determined time-points post-bolus administration of Clinical Center Reference Endotoxin 2 ng/kg (median with IQR, n = 10). Participants were asked to score the severity of headache, shivering, muscle ache, nausea on visual analogue scales (0 - 10, max score 40). Please click here to view a larger version of this figure.

'Low-dose' endotoxin in contrast has a much more diverse effect on individuals and may be used to explore inter-individual differences in the inflammatory response^31^. Administration of 0.06 - 0.2 ng/kg via either bolus or infusion induces 2 - 10 fold increases in plasma cytokines commensurate with those seen in chronic low-grade inflammatory conditions^32^. Such protocols may have a key role in exploring the contribution of inflammation and the pathways that regulate it to multiple complex cardiometabolic conditions^27^.

## Discussion

As demonstrated here and fully described in recent review articles, human IV endotoxin challenge is a unique experimental platform providing insight into the inflammatory pathways that underlie a vast range of human disease. Allowing the controlled, reproducible induction of systemic inflammation, the model permits access to the initial phases of the inflammatory cascade, eliminating potential confounding factors.

Participant's responses may be modulated with respect to severity and duration, and the genomic influence on their phenotypic presentation evaluated. Furthermore, the model may act as a test-bed for therapeutics, not only those against the LPS ligand itself, but LPS-induced signaling pathways and mediators of both the acute and resolution phases of inflammation. This may include agents targeted at restoration of endotoxin tolerance, a hyporesponsive state of the immune system that bears similarities to critical illness-induced immunoparalysis^33,34^ Able to replicate key features of both acute^16^ and chronic^27^ inflammation at not only a physiological/functional level but also a transcriptomic one^35^, IV endotoxin challenge seemingly has a key role to play in the translation of basic scientific breakthroughs to clinical practice. When coupled to directed investigation in appropriate animal models able to more closely replicate the pathophysiological responses of specific disease states (*e.g.*, murine cecal ligation and puncture and sepsis) this investigational paradigm may be especially powerful.

Safety of participants in any healthy human volunteer model is paramount. IV endotoxin challenge has been administered in the higher dose range of 2 - 4 ng/kg to thousands of individuals with, to our and other authors knowledge, no serious or permanent adverse events (personal communication, Dr. Anthony Suffredini)^19^. Of note however isolated accounts of severe vagal reactions have been reported^36,37^. The etiology presumably represents high resting vagal tone, volume depletion after overnight fasting, and catecholamine release with the onset of fever, chills and symptoms, culminating in an exaggerated Bezold-Jarisch reflex. This risk may be ameliorated by excluding individuals with previous vasovagal syncopy or a positive tilt-test, and volume loading with intravenous fluids prior to and during the endotoxin challenge^37^. Researchers employing high-dose endotoxin challenge (especially 4 ng/kg) should be cogent of the risk of bradycardia and/or cardiac pauses, warn participants of their rare but potential occurrence, employ appropriate monitoring, and have resuscitation equipment readily available. Other exclusion criteria, in addition to those described in the protocol, may be required dependent on experimental question. For instance, whilst it is prudent to exclude those that have participated in other clinical trials or experienced surgery/trauma in the past three months, restriction of entry (or use of a parallel-groups not a cross-over design in interventional studies) to those that have previously partaken in IV endotoxin challenge trials may also be required if immunological response is to be assessed: endotoxin tolerance persisting for an unknown length of time *in vivo*^38,39^.

Several limitations are apparent with the model. It is traditionally undertaken in healthy, young male volunteers un-representative of the clinical population. No interventions are required for the phenotypic consequences of endotoxin administration to resolve. Purified LPS injection provides exposure to only a single TLR ligand, as opposed to several immunogenic moieties on a live pathogen. By ethical necessity only a relatively modest inflammatory response may be elicited. Mimicry of every pathophysiological characteristic feature of disease is not achieved^40^. Thus 'any expectation that the model fully replicates the clinical condition of severe, localized or systemic gram-negative infection is un-warranted'^18^.

We would argue however that the model's strength lies not in inappropriate extrapolation to the clinical setting, but in interrogating the physiological, hematological, immunological, neuroendocrine and metabolic response of competent cells and organ systems to a key inflammatory stimulus. Thoughtful interpretation of the transient alterations provoked by endotoxin administration and their modulation by pharmacological challenge has been, and will continue to be, informative in designing new therapies and predicting their efficacy.

## Disclosures

The authors have nothing to disclose.
